# Sleep and Cardiovascular Health Among Women With a History of Hypertensive Disorders of Pregnancy: Pilot Observational Study

**DOI:** 10.2196/81118

**Published:** 2026-05-26

**Authors:** Anais Hausvater, Elianna Shwayder, Marie-Pierre St-Onge, Linda G Kahn, Manuela Plazas-Montana, Lillian Na, Amanda Joa, Leonardo Trasande, Harmony R Reynolds

**Affiliations:** 1 Sarah Ross Soter Center for Women's Cardiovascular Research, Cardiovascular Clinical Research Center, Leon H Charney Division of Cardiology, Department of Medicine, NYU Grossman School of Medicine, 550 First Ave, New York, NY, 10016, United States, 1 6465012828; 2 Department of Medicine, Brigham and Women's Hospital, Boston, NY, United States; 3 Division of General Medicine and Center of Excellence for Sleep and Circadian Research, Columbia University Irving Medical Center, New York, NY, United States; 4 Department of Pediatrics, New York University Grossman School of Medicine, New York, NY, United States; 5 Department of Population Health, New York University Grossman School of Medicine, New York, NY, United States

**Keywords:** sleep health, sleep quality, postpartum, hypertensive disorders of pregnancy, digital health, cardiovascular health

## Abstract

**Background:**

Both poor sleep health and hypertensive disorders of pregnancy (HDP) are independent risk factors for cardiovascular disease. Whether poor postpartum sleep contributes to the relationship between HDP and future cardiovascular disease is unknown. This pilot study evaluated the feasibility and acceptability of studying sleep health using a wearable device (Oura ring) among mothers of young children.

**Objective:**

We evaluated indices of sleep health both objectively with the Oura ring and subjectively via questionnaires and qualitative interviews among mothers with and without a history of HDP. We also aimed to compare cardiovascular health (CVH) among mothers with vs without a prior history of HDP.

**Methods:**

Women who were 3 to 7 years after childbirth completed baseline questionnaires (the Pittsburgh Sleep Quality Index [PSQI], Mediterranean Eating Pattern for Americans tool, and 7-item International Physical Activity Questionnaire) and wore the Oura ring continuously for 2 weeks to monitor sleep. Optimal sleep health was defined as a sleep duration of ≥7 hours, a PSQI score of ≤5, sleep timing with a sleep midpoint between 2 AM and 4 AM, a sleep efficiency of >85%, and a sleep onset variability of <60 minutes. CVH was assessed using the Life’s Essential 8 score, with 8 factors assessed via questionnaires (diet, physical activity, and nicotine exposure) and objective measurements (BMI, blood pressure, blood lipids, blood glucose, and sleep duration). Semistructured interviews were conducted.

**Results:**

In total, among 49 women, 28 (57%) with prior HDP and 21 (43%) with prior normotensive pregnancy were included, with an average of 4.9 (SD 1.2) years after delivery. Average sleep quality was suboptimal in both groups (mean PSQI score 7.0, SD 3.5 in the HDP group vs mean PSQI score 5.9, SD 2.4 in the control group; *P=*.22). Average sleep duration was suboptimal (6.7, SD 0.8 hours), with no difference between groups. Approximately half (n=23, 47%) had abnormal sleep timing, which was more common among those with a prior normotensive pregnancy. Sleep onset variability was high (mean 1.2, SD 0.5 hours), with no significant differences by HDP status. The mean CVH score fell within the moderate range (70.7, SD 12.8), with no differences between groups. The components of the CVH score that were lowest (ie, worst) among the entire cohort were diet (mean 37.3, SD 25.6) and BMI (mean 50.8, SD 35.4 kg/m^2^). Common barriers to sleep included parenting, work, and household responsibilities. The study met our criteria for the feasibility and acceptability of using the Oura ring to study sleep in this population.

**Conclusions:**

Among postpartum women, sleep health was suboptimal regardless of HDP history. Interventions to improve sleep and CVH should target all mothers during the first decade after childbirth.

## Introduction

Hypertensive disorders of pregnancy (HDP), including preeclampsia and gestational hypertension, complicate up to 15% of all pregnancies and are associated with significant maternal morbidity and mortality from cardiovascular disease (CVD) at the time of pregnancy and years after pregnancy [1-4]. Patients with a history of HDP are more likely to develop cardiovascular risk factors in the decade after delivery, including persistent hypertension and metabolic syndrome [5,6]. HDP are also associated with more than twice the risk of premature atherosclerosis and cardiovascular death, independent of other cardiovascular risk factors [7-9]. The concept of cardiovascular health (CVH) refers to a combination of modifiable factors (such as blood pressure [BP], diet, and physical activity) that, when optimized, contribute to a reduced risk of CVD. Women with prior HDP have worse CVH in the years after pregnancy than women with prior normotensive pregnancies, which is associated with an increased likelihood of subclinical CVD [10].

Sleep health is a multidimensional concept that includes several sleep indices such as duration (total sleep), efficiency (time spent asleep while in bed), timing, regularity, and quality (subjective assessment of satisfaction) [11,12]. Poor sleep health is common in US adults, is associated with cardiometabolic derangements (eg, increased adiposity [13], inflammation, and endothelial dysfunction), and predicts future cardiovascular events [13-18]. In 2022, adequate sleep duration of 7 to 9 hours per night was recognized by the American Heart Association (AHA) as a critical factor for CVH and was added to its aggregate measure of CVH, Life’s Essential 8 (LE8) [16,19]. Accordingly, women are particularly susceptible to poor CVH, as they report more sleep difficulties and insomnia symptoms than men, and poor sleep is more strongly associated with CVD among women than among men [20-26]. Mothers of young children are especially at risk of poor sleep, even beyond the immediate postpartum period [27]. Short sleep during pregnancy that persists for years after childbirth was associated with an increased risk of incident metabolic syndrome [28].

Poor sleep health during pregnancy is an independent risk factor for the development of HDP [29,30]. In the years after pregnancy, small studies have shown that women with prior HDP report lower sleep quality and shorter sleep duration [31,32]. Poor sleep was more common among those with prior HDP and persistent hypertension than among those in whose BP had normalized after childbirth [33]. Women with prior preeclampsia reported poorer sleep quality and frequent sleep disturbances than women with healthy pregnancy controls 1‐5 years after childbirth, which were associated with higher 24-hour BP [34].

The Oura ring (version Gen3; Oura Health Inc) is a wearable device that uses infrared photoplethysmography, a temperature sensor, and a 3D accelerometer to measure data such as heart rate, respiration, activity, movement, and body temperature. The ring has been successfully used in prior studies focused on women’s health but has never been specifically studied among postpartum women with prior HDP [35,36]. The Oura ring is paired with a mobile app that provides users with daily feedback related to sleep health. Validation studies comparing the Oura ring with gold-standard polysomnography and research-grade actigraphy are small but have shown acceptable correlation for sleep duration and architecture [37-39]. Given the widespread commercial use of wearable devices such as the Oura ring, it is essential that we better understand their utility and potential applications in future interventions aimed at optimizing health.

The goal of this pilot study was to evaluate the use of a novel and commercially available wearable device, the Oura ring, to measure sleep health among mothers with and without a history of HDP and to better understand subjective barriers and facilitators to sleep health in this population. We also aimed to compare CVH among mothers with vs without a prior history of HDP and to evaluate how sleep health relates to the other components of CVH.

## Methods

### Study Participants

We performed a nested cohort pilot study examining sleep characteristics among women who were 3 to 7 years after childbirth. We recruited participants enrolled in the New York University (NYU) Children’s Health and Environment Study (CHES). The methods of CHES have been previously described [40]. In brief, CHES is a prospective cohort study that recruits pregnant women aged ≥18 years who are at <18 weeks of gestation from prenatal visits at obstetrics clinics at NYU Langone-Manhattan, NYU Langone-Brooklyn, and Bellevue Hospital. The postnatal phase of CHES includes follow-up of mothers during their children’s scheduled study visits (see Multimedia Appendix 1 for interview guide).

This study included CHES participants with scheduled 3 to 5 and 5 to 7 years of follow-up study visits. To account for expected sleep disturbances in the perinatal and postpartum periods, we excluded women who were currently pregnant or within 1 year after childbirth. Our cases included women with a history of HDP, defined as preeclampsia, superimposed preeclampsia, or gestational hypertension in any prior pregnancy. HDP was ascertained by manual chart review and diagnosis codes and was confirmed by participant self-report. Controls were CHES participants with a history of uncomplicated, normotensive pregnancies free of complications such as gestational diabetes or preterm birth who were attending scheduled follow-up study visits during the same time frame as cases.

During study visits, participants had their weight and BP measured and were given an Oura ring, a wearable sleep-tracking device, which they were asked to wear continuously for 2 weeks. Participants had access to the Oura mobile phone app, which provides daily statistics on sleep duration, efficiency, and timing. Participants also completed diet, physical activity, and sleep questionnaires, including the Pittsburgh Sleep Quality Index (PSQI), Mediterranean Eating Pattern for Americans tool, and the 7-item International Physical Activity Questionnaire short form, and reported nicotine exposure. Blood samples were taken from participants (nonfasting) and analyzed for blood lipids and hemoglobin A_1c_ (a measure of blood glucose).

After the 2-week study period, select participants were invited to complete semistructured interviews via video conference. Purposive sampling was used to select participants with diverse races or ethnicities, languages, and sleep scores. They were asked questions related to barriers and facilitators to sleep health, as well as components of a sleep intervention that they thought might be feasible.

### CVH Score

Participant CVH was assessed using the AHA LE8 scoring system, which includes the following factors: diet, physical activity, nicotine exposure, BMI, BP, blood lipids, blood glucose (hemoglobin A_1c_), and sleep duration [16]. Each factor is scored from 0 to 100 based on the AHA scoring system, and the total score is an unweighted average. CVH is categorized as high (80-100), moderate (50-79), or low (0-49) [16]. Physical activity data collected using the 7-item International Physical Activity Questionnaire short form were converted to minutes per week of moderate or vigorous physical activity and mapped to the AHA LE8 scoring algorithm to derive a physical activity score from 0 to 100 [16]. If any components of the CVH score were missing, the overall score was averaged based on the number of components available (see Multimedia Appendix 2 for study questionnaires).

### Sleep Health

Sleep health was measured objectively via the Oura ring wearable device and subjectively using the PSQI. Poor sleep was assessed based on 5 metrics: sleep duration (short sleep defined as <7 hours), sleep efficiency (low defined as <85%), sleep timing (suboptimal defined as a sleep midpoint before 2 AM or after 4 AM), sleep variability (elevated defined as an SD of nightly bedtime >60 minutes), and sleep quality (poor quality defined as PSQI >5) [11].

### Statistical Analyses

Categorical variables, including demographic characteristics, medical history, and binary sleep measures (eg, abnormal sleep timing), were compared using the Fisher exact test. Continuous variables, including age, parity, CVH scores and their components, and continuous sleep metrics, were compared using Student *t* tests or Mann-Whitney *U* tests, as appropriate based on distribution. To evaluate whether age or current hypertension status explained observed between-group differences, we fit 3 separate regression models for each sleep outcome: an unadjusted model including group (HDP vs control), a model adjusted for age, and a model adjusted for current hypertension status, using linear models for continuous sleep outcomes and logistic regression for abnormal sleep timing. We performed Spearman correlation testing between sleep duration and the other 7 CVH component scores. Feasibility of using the Oura ring sleep tracker in this population was measured based on compliance with the Oura ring (percentage of hours worn over the 2-week period). Our feasibility target was met if >85% of participants wore the Oura ring for >70% of nights. Acceptability was measured using the Acceptability of Intervention Measure (AIM), a 4-item measure of perceived intervention acceptability. AIM items are measured on a 5-point Likert scale (completely disagree–completely agree), and the score for each item is calculated as the mean [41]. There is no universal cutoff for AIM scores. Higher AIM scores indicate a more acceptable intervention. On the basis of prior literature, we defined acceptability as an average score of ≥4 on each AIM item. Qualitative interviews were analyzed using thematic content analysis and coded using ATLAS.ti (version 25; Lumivero LLC) software. ES coded the interviews, and emergent themes were thoroughly discussed with AH.

### Ethical Considerations

The study was approved by the NYU Grossman School of Medicine Institutional Review Board (i23-00046), and all participants provided written informed consent before participation. All study data were deidentified to protect the privacy and confidentiality of participants. All participants were compensated with a US $75 Amazon gift card for participation in the study.

## Results

### Participant Characteristics

In total, 49 participants completed the study. Of these, (n=28, 57%) had a prior delivery complicated by HDP and 21 (43%) had a prior uncomplicated pregnancy (Table 1). Assessments occurred an average of 4.9 (SD 1.2) years after pregnancy. This was a diverse cohort, with 29% (n=14) of women identified as White, 14% (n=7) as non-Hispanic Black, and 67% (n=33) as Hispanic. Women with prior HDP were older and more likely to have chronic hypertension (Table 1). There were otherwise no baseline differences in medical comorbidities between groups. Of the HDP group with 28 women, 7 (25%) had pre-existing hypertension with superimposed preeclampsia, 18 (64%) had preeclampsia, and 3 (11%) had gestational hypertension.

**Table 1.** Baseline characteristics.

Variables	All participants (n=49)	Participants with a history of hypertensive disorders of pregnancy (n=28)	Participants with a history of normotensive pregnancy (n=21)	*P* value
Age (years), mean (SD)	35.5 (6.9)	37.2 (6.8)	33.1 (6.4)	.04
Race, n (%)				.37
Asian	4 (8)	4 (14)	0 (0)	
Black or African American	7 (14)	4 (14)	3 (14)	
Other	23 (47)	12 (43)	11 (52)	
Unknown	1 (2)	0 (0)	1 (5)	
White	14 (29)	8 (29)	6 (29)	
Ethnicity, n (%)				.66
Hispanic or Latinx	33 (67)	17 (60)	16 (76)	
Not Hispanic or Latinx	15 (31)	10 (36)	5 (24)	
Unknown	1 (2)	1 (4)	0 (0)	
Marital status, n (%)				.24
Married or living with a partner	42 (86)	17 (60)	16 (76)	
Divorced or separated	2 (4)	10 (36)	5 (24)	
Single or widowed	5 (10)	1 (4)	0 (0)	
Parity, median (IQR)	2.0 (2.0-3.0)	2.0 (2.0-3.0)	2.0 (1.8-3.0)	.43
Education, n (%)				.36
High school or less	24 (49)	12 (43)	12 (57)	
Some college but no degree	7 (14)	3 (11)	4 (19)	
Associate degree	2 (4)	1 (4)	1 (4)	
Bachelor’s degree	8 (16)	6 (21)	2 (10)	
Postgraduate degree	8 (16)	6 (21)	2 (10)	
Insurance, n (%)				.02
Public	36 (73)	17 (61)	19 (90)	
Private	13 (27)	11 (39)	2 (10)	
Medical history, n (%)				
Obesity (BMI ≥30 kg/m^2^)	25 (51)	13 (46)	12 (57)	.57
Hypertension	14 (29)	13 (46)	1 (5)	<.001
Diabetes	4 (8)	3 (11)	1 (5)	.68
High cholesterol	5 (10)	2 (7)	3 (14)	.64
Obstructive sleep apnea	2 (4)	1 (4)	1 (5)	1

### Feasibility and Acceptability

We consented 52 participants and accrued 49 participants for this study over 12 months. Overall, 3 women did not initiate the study due to technological challenges. All included participants had a minimum of 7 nights of data. Of the 49 participants, 90% (n=44) wore the Oura ring for >70% of nights over the 2-week study period, indicating excellent adherence. Our acceptability target was met (median AIM item score 4, IQR 4-5; Table S1 in Multimedia Appendix 3). Participants’ feedback on the ring-wearing experience was generally positive, including: “It (Oura ring) is very easy to use,” “(Oura ring is) a great device,” and “It (Oura app) helped me realize that I don’t sleep well.”

### Sleep Health

**Figure 1.**
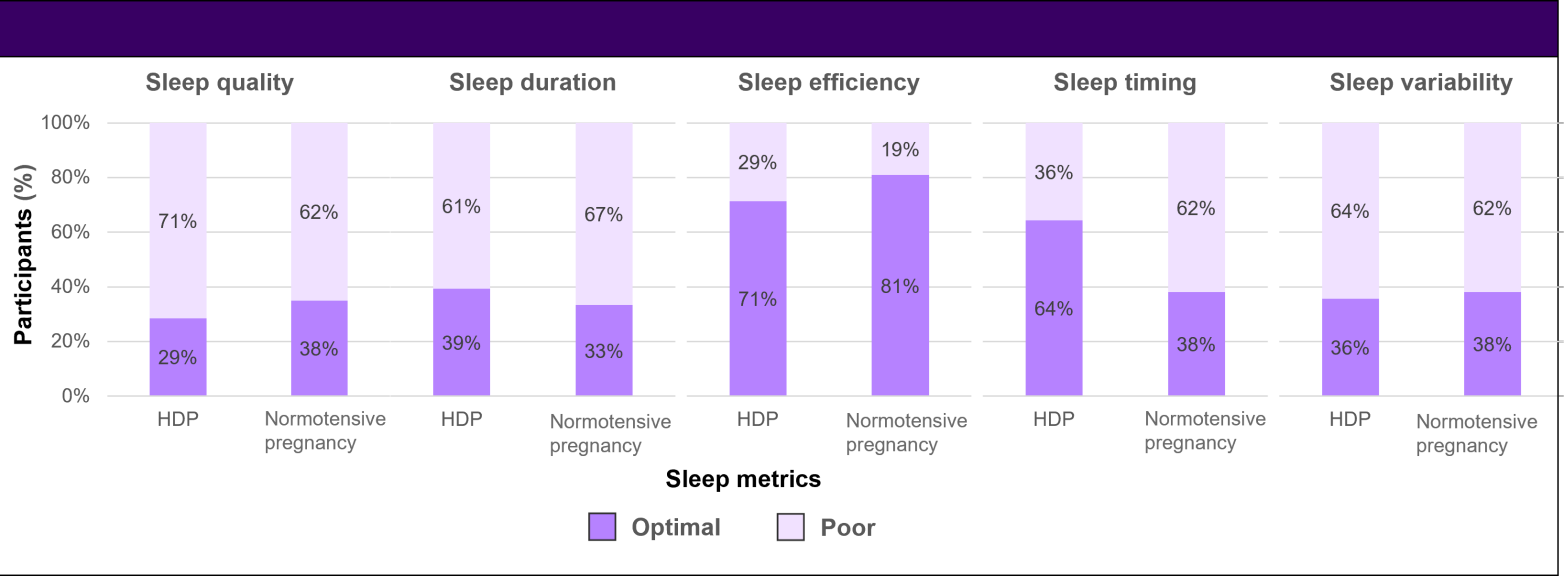
Sleep metrics among women with and without a history of hypertensive disorders of pregnancy (HDP). Optimal sleep quality was defined as a Pittsburgh Sleep Quality Index score of ≤5. Optimal sleep duration, efficiency, timing, and variability were derived from Oura ring data and defined as follows: duration ≥7 hours per night, sleep efficiency ≥85%, sleep timing with a sleep midpoint between 2 AM and 4 AM, and sleep variability defined as an SD in bedtime <1 hour.

In the unadjusted analysis, there were no differences in the proportion of women who achieved optimal sleep quality, efficiency, duration, timing, or regularity when comparing those with vs without prior HDP (Table 2, Figure 1). Sleep quality was suboptimal on average across the cohort and was similar among HDP and control participants (mean PSQI score 7.0, SD 3.5 in the HDP group vs mean PSQI score 5.9, SD 2.4 in the control group; *P=*.22) [Table 2]. Oura ring-derived sleep data revealed that average sleep duration was suboptimal for most mothers (mean 6.7, SD 0.8 hours), with no difference between HDP and control participants (*P=*.99). Sleep timing was abnormal in 47% of participants overall. Sleep onset variability was high on average across the entire cohort, reflecting suboptimal regularity in bedtimes (mean 1.2, SD 0.5 hours), but was similar between groups (*P=*.97). Sleep efficiency was normal overall and did not differ between groups (mean 86.9%, SD 3.8%; *P=*.62) [Table 2]. Approximately one-third of participants met criteria for optimal sleep across individual domains.

**Table 2.** Sleep outcomes.

Sleep outcomes	All participants (n=49)	Participants with a history of hypertensive disorders of pregnancy (n=28)	Participants with normotensive pregnancy (n=21)
Sleep duration (hours), mean (SD)	6.65 (0.80)	6.65 (0.75)	6.66 (0.83)
Sleep efficiency (%), mean (SD)	86.9 (3.83)	86.65 (4.27)	87.21 (3.24)
Abnormal sleep timing, n (%)	23 (47)	10 (36)	13 (62)
Sleep variability (hours), mean (SD)	1.22 (0.52)	1.22 (0.60)	1.21 (0.41)
Sleep quality (Pittsburgh Sleep Quality Index), mean (SD)	6.49 (3.09)	6.96 (3.48)	5.86 (2.41)

In regression analyses, HDP was not associated with sleep duration, sleep efficiency, sleep quality (PSQI), continuous sleep timing, or sleep variability in either unadjusted or age-adjusted models; findings were similarly nonsignificant in models adjusted for baseline hypertension (Table 3). After adjustment for current hypertension status, abnormal sleep timing—defined as a sleep midpoint outside the interval of 2 AM to 4 AM—was more common among those with prior normotensive pregnancy (Table 3). The corresponding unadjusted and age-adjusted estimates showed a trend toward more abnormal sleep timing among those with prior normotensive pregnancy (both *P*≈.07).

**Table 3.** Linear regression estimates comparing sleep metrics between participants with a history of hypertensive disorders of pregnancy (HDP) and participants with normotensive pregnancy (control).^a^

Sleep metrics	β coefficient^b^	95% CI	*P* value
Sleep duration			
Unadjusted	0	−0.46 to 0.46	.99
Adjusted for age	0.15	−0.32 to 0.61	.53
Adjusted for hypertension	0.22	−0.28 to 0.72	.39
Sleep efficiency			
Unadjusted	−0.56	−2.80 to 1.68	.62
Adjusted for age	0.31	−1.93 to 2.55	.78
Adjusted for hypertension	0.74	−1.66 to 3.15	.54
Abnormal sleep timing (continuous)			
Unadjusted	−0.66	−1.45 to 0.14	.10
Adjusted for age	−0.53	−1.37 to 0.31	.21
Adjusted for hypertension	−0.69	−1.59 to 0.22	.13
Sleep variability			
Unadjusted	0.01	−0.30 to 0.31	.97
Adjusted for age	−0.03	−0.35 to 0.30	.88
Adjusted for hypertension	−0.16	−0.49 to 0.17	.32
Sleep quality (Pittsburgh Sleep Quality Index score)			
Unadjusted	1.11	−0.68 to 2.89	.22
Adjusted for age	1.28	−0.61 to 3.17	.40
Adjusted for hypertension	0.33	−1.63 to 2.30	.11
Abnormal sleep timing (binary)			
Unadjusted	−1.07	−2.28 to 0.08	.07
Adjusted for age	−1.14	−2.44 to 0.08	.07
Adjusted for hypertension	−1.52	−3.04 to −0.18	.03

a Values are presented as β coefficients representing the adjusted mean difference (HDP − control) for linear regression and log-odds for logistic regression, with 95% CIs and *P* values shown for unadjusted models and models adjusted for age or current hypertension.

b β coefficients represent mean differences for all outcomes except abnormal sleep timing (binary), for which β coefficients represent log-odds.

Optimal sleep quality was defined as a PSQI score of ≤5. Optimal sleep duration, efficiency, timing, and variability were derived from Oura ring data and defined as follows: sleep duration ≥7 hours per night, sleep efficiency ≥85%, sleep timing with a sleep midpoint between 2 AM and 4 AM, and sleep variability defined as an SD in bedtime <1 hour.

### CVH Score Analysis

**Figure 2.**
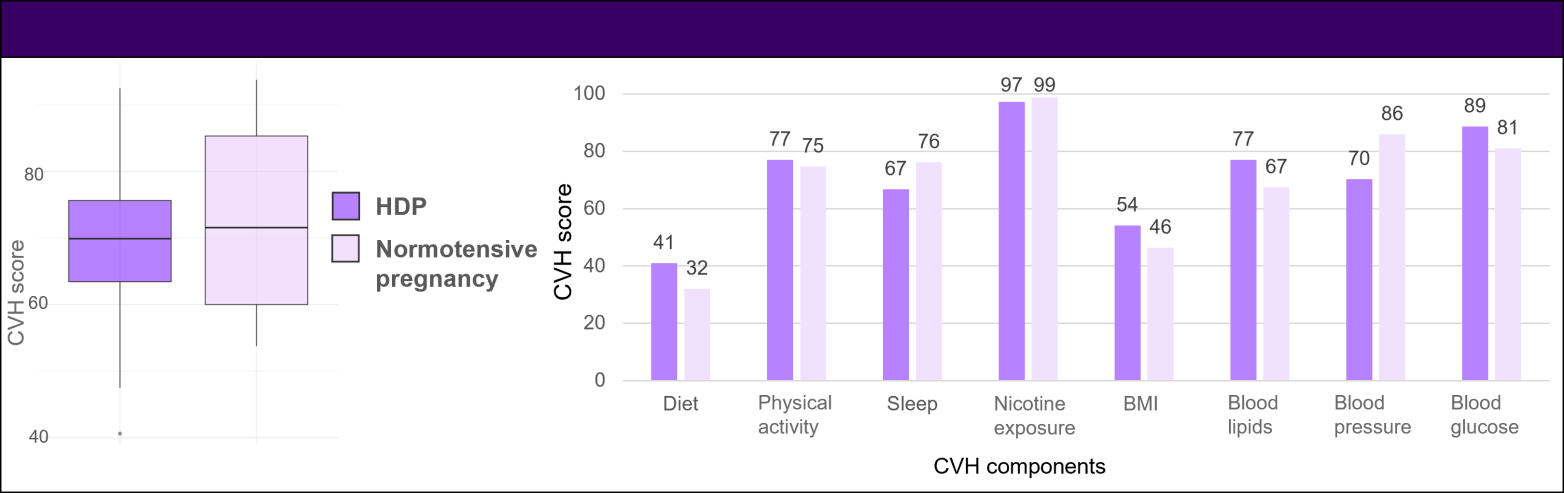
Cardiovascular health (CVH) scores among women with and without a history of hypertensive disorders of pregnancy (HDP). The CVH score reflects the unweighted average of 8 components: diet, physical activity, sleep, nicotine exposure, BMI, blood lipids, blood pressure, and blood glucose. Each component was scored from 0 to 100 based on the American Heart Association Life’s Essential 8 criteria. Higher scores indicate better health.

AHA CVH score was in the moderate range overall (mean 70.77, SD 12.88), with no difference between groups (mean 70.9, SD 11.4 vs mean 70.3, SD 14.7; *P=*.87; Figure 2, Table S2 in Multimedia Appendix 3). The components of the CVH score that were lowest (ie, worst) among the entire cohort were diet (mean 37.3, SD 25.6) and BMI scores (50.8, SD 35.4; Table S2 in Multimedia Appendix 3). There were no differences in any components of the CVH score between those with vs without prior HDP (Figure 2, Table S2 in Multimedia Appendix 3), but there was a trend toward lower scores (ie, higher BPs) among the group with prior HDP that was not statistically significant (*P*=.07; Table S2 in Multimedia Appendix 3). In the full cohort, there were no significant correlations between the CVH component score for sleep health and the other 7 components of LE8 (Table S3 in Multimedia Appendix 3).

### Patient Perspectives

In total, 19 (39%) participants were invited to complete qualitative interviews (PSQI scores range 1-14; n=19, 42% Hispanic). Of these, 14 (74%) participants consented and were interviewed about their experiences after the study period (PSQI scores 1-14; n=14, 36% Hispanic). Of the 14 participants, 12 (86%) had prior HDP. All participants noted that tracking their sleep metrics had some influence on them, ranging from increased awareness of poor sleep habits to active modification of behaviors related to bedtime, sleep duration, or sleep hygiene.

Only 2 (14.3%) participants described a dedicated bedtime routine including habits explicitly intended to facilitate sleep, such as avoiding screens, maintaining a consistent bedtime, drinking tea, taking a bath, and reading. Overall, 6 (42.9%) participants identified sleep-facilitating practices that they engaged in more sporadically, such as meditating, reading, drinking tea, and using scented oils. Only 3 (21.4%) participants reported routines centered around their children’s bedtime, including reading, singing, and cuddling. Only 3 (21.4%) participants lacked a consistent bedtime routine. Overall, 11 (78.6%) participants described behaviors and responsibilities that adversely impacted sleep, including late-night screen time (n=8, 57.1%), inconsistent bedtimes (n=6, 42.9%), late-night parenting duties (n=6, 42.9%), working late (n=6, 42.9%), and household chores (n=2, 14.3%). The remaining 3 (21.4%) participants did not identify specific activities that hindered sleep.

Barriers to adequate sleep were thematically related to societal demands, family responsibilities, and individual factors. Societal demands included competing work, domestic, and caregiver responsibilities, and gender-related norms such as the burden of childcare falling on the mother (eg, child health- or school-related challenges and the “mental load” of parenting). Financial stressors and environmental disturbances (eg, city noise) were also identified as external factors that negatively impacted sleep. Themes centered on the family unit included parenting roles (eg, child-centered bedtime routines), dynamics with partners (eg, maintaining relationship with a significant other), and child-specific barriers (eg, cosleeping and child sleep difficulty). Individual barriers included a desire for personal time (often only available after children were asleep), a lack of prioritization of sleep health in general, a lack of consistency with sleep-facilitating activities, being a light sleeper, high stress levels, and waking during the night.

When prompted to consider modifiable aspects of their sleep routine, 7 (50%) participants favored bedtime consistency, 4 (28.6%) participants wanted to sleep 7 to 8 hours every night, 1 (7.1%) participant viewed both approaches as feasible, and 2 (14.3%) participants felt their sleep habits were not modifiable. When asked what type of messaging might motivate mothers to engage in a sleep intervention, 6 (42.9%) participants believed that motherhood-focused messages would be most salient (“If I had better sleep habits, I’d be more aware of my child and be a better mother”), 3 (21.4%) participants favored emphasizing personal health (“If you sleep enough, you’re more productive and gain health benefits”), and 2 (14.3%) participants advocated for emphasizing the intertwined nature of personal health, parenting, and quality of life (“Improving your health means staying around longer for your kids, modeling good habits, and being less tired to care for them”). The remaining 3 (21.4%) participants did not identify a specific motivating messaging approach or expressed uncertainty about what type of messaging would influence their behavior.

## Discussion

### Principal Findings

In this pilot study, the Oura ring wearable device was a feasible and acceptable tool for measuring sleep in this population of mothers of young children. Mothers of children aged 3 to 7 years had suboptimal sleep quality, duration, and regularity, independent of prior HDP status. Barriers to sleep health in this population included parenting, professional, and domestic responsibilities. Most participants lacked a consistent bedtime routine and regularly engaged in behaviors that adversely affect sleep. Most participants cited that tracking their sleep with the Oura ring inspired awareness of poor sleep health. These data provide support for the development of future sleep optimization intervention studies aimed at optimizing CVH.

In regression analyses, there was a significant association between abnormal sleep timing and prior normotensive pregnancy in the model adjusted for current hypertension status. This finding was not observed in unadjusted or age-adjusted models. Therefore, adjustment for current hypertension may represent overadjustment, as chronic hypertension could lie on the causal pathway between HDP and later sleep or CVH. As such, this result is considered exploratory and hypothesis-generating and requires confirmation in larger, adequately powered cohorts.

### Prior Work

To our knowledge, this is the first study to evaluate sleep indices objectively using a wearable device in a population of mothers within the decade after delivery. Our findings are in line with prior studies showing that maternal sleep is poor years after delivery [27,42]. Insomnia and short sleep (<7 hours per night) are common among mothers in the first decade after delivery [28,42]. Sleep duration, as measured by the Oura ring, was overall worse when compared with a cohort of healthy young and midlife women from the United States [43].

Previous studies that relied solely on subjective sleep metrics found that years after pregnancy, women with prior HDP report poor sleep quality and short sleep duration [31,32]. In one such study of 526 women, 20% of whom had HDP, the PSQI was completed to evaluate their sleep quality an average of 13 years after delivery. Sleep quality scores were poor overall and were slightly worse among women with prior HDP than those with normotensive pregnancies (6.6 vs 5.8; *P=*.04) [31]. Similar to this study’s analysis, the investigators found that sleep duration was suboptimal but did not differ between groups [31]. A small study of 13 women with prior preeclampsia reported poorer sleep quality and more frequent sleep disturbances than healthy pregnancy controls 1 to 5 years after childbirth, and these sleep problems were associated with higher 24-hour BP [34]. In this study, we found that certain metrics, such as sleep timing and some CVH metrics, including diet, BMI, and blood lipids, were numerically worse among participants with prior normotensive pregnancies. This might be explained by the small numbers of participants in each group, as well as differences in socioeconomic and cultural factors between groups. The control arm had a higher proportion of Hispanic participants, lower levels of education, and a higher proportion of public insurance. These differences in socioeconomic factors alone may lead to poor sleep and CVH. Taken together, sleep optimization strategies may benefit all mothers in the first decade after delivery and may be of particular importance among those at higher cardiovascular risk and lower socioeconomic status.

To our knowledge, ours is the first study to combine both objective and subjective sleep data in this population, with the aim of capturing the full spectrum of sleep health. Prior literature suggests that subjective measures of sleep health can overestimate or underestimate metrics such as sleep duration, efficiency, and sleep timing [44]. There is poor correlation between perceptions of sleep as “poor” or “sufficient” and objective sleep measures of sleep duration and depth [44]. Individual perceptions of sleep quality and daytime functioning are subjective measures of sleep health that provide different but equally essential information. Thus, both objective and subjective sleep data are required to fully understand the whole spectrum of a person’s sleep health.

Despite the growing recognition of sleep health as an important contributor to cardiovascular risk, most physicians fail to routinely inquire about a patient’s sleep health or counsel patients on how to improve sleep [45]. This pilot study demonstrates that it is feasible to track sleep health using a wearable device among mothers of young children. Importantly, sleep tracking with the Oura ring heightened participants’ awareness of their suboptimal sleep health, and mothers were receptive to feedback regarding their sleep behaviors. This increased awareness underscores an unmet need for patient education on the connection between sleep health and cardiovascular risk and lifestyle measures that can improve CVH. This education may be particularly pertinent for women with prior HDP, given their limited awareness of elevated cardiovascular risk [46]. Once informed, women with a history of HDP express a strong desire for clinician guidance to support long-term lifestyle change [46]. Similarly, in our cohort, participants discussed that the sleep data they received from the Oura ring helped them identify feasible behavioral targets, namely consistent bedtime and increased sleep duration. The potential for wearable sleep trackers such as the Oura ring to facilitate durable improvements in sleep behavior remains uncertain. Digital health interventions, including wearable devices, may improve cardiovascular risk factors such as BP, physical activity, and weight among postpartum women, but studies to date have been small and have shown mixed results [39].

### Strengths and Limitations

This is the first study to examine the feasibility and acceptability of using the Oura ring, a popular commercial device, to measure sleep health in mothers of young children. However, there are several limitations worth noting. First, this pilot study was not sufficiently powered to detect clinically meaningful differences in sleep health and CVH components between groups. However, these data may be used to estimate variance for powering larger observational studies. Second, we only monitored sleep for 2 weeks, which may not have captured the full spectrum of participants’ sleep habits. To mitigate this limitation, participants were instructed to wear the ring during periods when they are following their habitual schedule (eg, not while on vacation). It is possible that sleep behaviors changed in response to wearing the Oura ring, but this is likely mitigated by the fact that we are reporting averages across the study period between both groups. Third, the Oura ring has been validated against research-grade actigraphy in only small-sized studies [37]. Fourth, we did not include screening for sleep apnea or insomnia, which limited our ability to differentiate sleep disorders from poor sleep health. Finally, this was a substudy of a larger observational cohort (CHES), which may have introduced selection bias.

### Future Directions

Our findings suggest that sleep and CVH optimization efforts should extend beyond women with prior HDP to a broader postpartum population in the first decade after delivery. Future studies in larger cohorts are necessary to determine whether wearable sleep-tracking interventions effectively support sustained behavioral change, particularly among mothers navigating the unique sleep-related challenges associated with early parenthood.

### Conclusions

In this cohort of postpartum mothers, we found that it was feasible and acceptable to use the Oura ring to study sleep among women years after delivery. We found that sleep quality, duration, and variability were uniformly poor, with no differences between those with and without prior HDP. CVH was suboptimal across all participants.

## Supplementary material

10.2196/81118 Multimedia Appendix 1 Qualitative interview guide.

10.2196/81118 Multimedia Appendix 2 Study questionnaires.

10.2196/81118 Multimedia Appendix 3 Additional study tables on Acceptability of Intervention Measure questionnaire results, cardiovascular health and its components, and correlations between the sleep health score and cardiovascular health components.

## Abbreviations

AHA: American Heart Association
AIM: Acceptability of Intervention Measure
BP: blood pressure
CHES: Children’s Health and Environment Study
CVD: cardiovascular disease
CVH: cardiovascular health
HDP: hypertensive disorders of pregnancy
LE8: Life’s Essential 8
NYU: New York University
PSQI: Pittsburgh Sleep Quality Index
