# Supplementary material for: Sleep and Cardiovascular Health Among Women With a History of Hypertensive Disorders of Pregnancy: Pilot Observational Study
Source: JMIR Cardio. 2026 May 26;10:e81118. doi: 10.2196/81118 (PMC13211945; doi:10.2196/81118)
Supplement: Multimedia Appendix 1 [file cardio-v10-e81118-s001.docx]

**Interview Guide**

- Do you have a usual bedtime routine?
  - [prompt] for example, clean your face, read, meditate, watch TV
  - If so, what is it like and how long is it?
  - If not, what prevents you from having a usual bedtime routine?
- What are some activities that help you fall asleep and stay asleep?
  - [prompt] for example: bath, reading, meditation, cold room
- What you do you think could help you sleep better?
  - [probe] tell me more about what might help you sleep better
- Do you have a usual bedtime within an hour every night?
  - [prompt] for example: every night you fall asleep between 10PM and 11PM
  - If so, what is the 1-hour timeframe you usually go to bed?
  - If not,
    - If you were asked to go to sleep around the same time every night, do you think this would be possible?
    - what are some reasons your bedtime might differ from night to night?
- Are you able to fall asleep easily once you are in bed?
  - [prompt] for example, do you fall asleep within 20 minutes of getting into bed?
  - If so, what does fall asleep easily mean to you?
  - If not, what prevents you from falling asleep?
    - [prompt] for example, are you kept up at night by factors such as stress, noise, light, disturbances from other people in the home, work etc.
- Do you get woken from sleep more than once per night?
  - If so, what wakes you up from sleep?
    - [prompt] for example do you get woken up by child disturbance, disturbance by others in the home, light, noise, stress, temperature, other discomforts?
      - [probe] tell me more about these disturbances- how often do they occur?
      - [probe] if woken up by your child [children]: how many children do you have and how old are they?
- How was your experience with the Oura ring?
  - [probe] Did tracking your sleep with the Oura ring change your sleep habits?
    - If so, how did it change your habits?
  - [probe] Did the information on the Oura app make a difference to your sleep?
    - If so
      - what information was most helpful?
      - how did knowledge about your sleep influence your thinking and/or behavior?
- If I were interested in testing a sleep program to try to improve your sleep, what do you think would be helpful to include?
  - [prompt] would access to information about your daily sleep with feedback be helpful?
    - [prompt] for example, wearing the Oura ring that tells you how well you slept
  - [prompt] would it be helpful to receive text messages with tips on how you could improve your sleep?
    - [probe] How often would you like to receive these texts?
  - [prompt] for example, text messages would give you hints on how to improve your sleep.
  - If you were asked to go to sleep around the same time every night, do you think this would be possible?
