# Supplementary material for: Sleep and Cardiovascular Health Among Women With a History of Hypertensive Disorders of Pregnancy: Pilot Observational Study
Source: JMIR Cardio. 2026 May 26;10:e81118. doi: 10.2196/81118 (PMC13211945; doi:10.2196/81118)
Supplement: Multimedia Appendix 2 [file cardio-v10-e81118-s002.pdf]

IPAQ Short

Please complete the survey below to the best of your abilities.

Thank you!

We are interested in finding out about the kinds of physical activities that people do as part of their everyday lives. The questions are about the time you spent being physically active in the last 7 days. Please answer each question even if you do not consider yourself to be an active person. In answering the following questions, vigorous physical activities refer to activities that take hard physical effort and make you breathe much harder than normal. moderate activities refer to activities that take moderate physical effort and make you breathe somewhat harder than normal.

1a. During the last 7 days, on how many days did you do vigorous physical activities like heavy lifting, digging, aerobics, or fast bicycling?

Think about only those physical activities that you did for at least 10 minutes at a time.

- ◯ 1 day per week
- ◯ 2 days per week
- ◯ 3 days per week
- ◯ 4 days per week
- ◯ 5 days per week
- ◯ 6 days per week
- ◯ 7 days per week
- ◯ none

1b. How much time in total did you usually spend on one of those days doing vigorous physical activities?

hours ____________________

minutes ____________________

2a. Again, think only about those physical activities that you did for at least 10 minutes at a time. During the last 7 days, on how many days did you do moderate physical activities like carrying light loads, bicycling at a regular pace, or doubles tennis? Do not include walking.

- ◯ 1 day per week
- ◯ 2 days per week
- ◯ 3 days per week
- ◯ 4 days per week
- ◯ 5 days per week
- ◯ 6 days per week
- ◯ 7 days per week
- ◯ none

2b. How much time in total did you usually spend on one of those days doing moderate physical activities?

hours ____________________

minutes ____________________

3a. During the last 7 days, on how many days did you walk for at least 10 minutes at a time? This includes walking at work and at home, walking to travel from place to place, and any other walking that you did solely for recreation, sport, exercise or leisure.

- ◯ 1 day per week
- ◯ 2 days per week
- ◯ 3 days per week
- ◯ 4 days per week
- ◯ 5 days per week
- ◯ 6 days per week
- ◯ 7 days per week
- ◯ none

3b. How much time in total did you usually spend walking on one of those days?

hours ______________________

minutes ______________________

The last question is about the time you spent sitting on weekdays while at work, at home, while doing course work and during leisure time. This includes time spent sitting at a desk, visiting friends, reading traveling on a bus or sitting or lying down to watch television.

4. During the last 7 days, how much time in total did you usually spend sitting on a week day?

hours ______________________

minutes ______________________

MEPA-16 Diet Questionnaire

Please complete the survey below to the best of your abilities.

Thank you!

Answer the following questions about how often you eat foods or drink beverages each day or each week. Answer based on the number of times you eat that food either in a day or week. The amount of food for one serving is listed in parentheses after each question. If you do not eat that food, choose the "less than" option.

---

35) How much olive oil do you consume per day? (Including that used in frying, meals eaten away from home, salads, etc.) ? (A serving is one tablespoon of olive oil)

- ◯ < 2 servings per day
- ◯ ≥ 2 servings per day

---

36) How many servings of green leafy vegetables do you consume per week?
(A serving is 1 cup raw or ½ cup cooked hardy greens like kale, collards, chard, or 2 cups leafy lettuce greens)

- ◯ < 7 servings per week
- ◯ ≥7 servings per week

---

37) How many servings of other vegetables do you consume per day?
(A serving is ½ cup of other vegetables. Specific examples like half a large bell pepper, 5-8 broccoli or cauliflower florets, 6 baby or 1 medium carrot, ½ large ear of corn, half a medium potato 2-3 inches across, half a large sweet potato, half a large zucchini, etc).

- ◯ < 2 servings per day
- ◯ ≥2 servings per day

---

38) How many servings of berries do you consume per week?
(A serving is 1 cup blackberries, blueberries, raspberries, halved strawberries, 12 fresh/frozen cherries)

- ◯ < 2 servings per week
- ◯ ≥2 servings per week

---

39) How many servings of other fruit do you consume per day?
(A serving is 1 medium whole fruit like apple, orange, peach or banana, 1 cup of cut up fruit like pineapple or melon, ½ cup grapes, mango, or dried fruit, 2 small tangerines, 2 tablespoons raisins)

- ◯ < 1 servings per day
- ◯ ≥1 servings per day

---

40) How many servings of red meat, hamburger, bacon or sausage do you consume per week?
(A serving is 3 ounces of red meat, pork, ground meat, ham (size of a deck of cards), 3 slices bacon, 1 hot dog or sausage)

- ◯ >3 servings per week
- ◯ ≤ 3 servings per week

---

41) How many servings of fish do you consume per week?
(A serving is 4 ounces of fin fish like salmon, cod, trout, herring (the size of a checkbook); 6 oysters or clams, 2 medium sardines, 10 medium shrimp or scallops)

- ◯ < 1 servings per week
- ◯ ≥1 servings per week

---

42) How many servings of chicken do you consume per week?
(A serving is 3 ounces or white or dark meat (size of a deck of cards)

- ◯ >5 servings of chicken per week
- ◯ ≤ 5 servings of chicken per week

43) How many servings of full fat or regular cheese or cream cheese do you consume per week?
(A serving is 1 ounce of cheese such as cheddar, American, Swiss, Monterey Jack, feta, mozzarella, ¼ cup cottage cheese, ¼ cup ricotta, 2 tablespoons parmesan, 1 tablespoon of cream cheese, 1.5 tablespoons of reduced fat cream cheese)

◯ >4 servings per week
◯ ≤ 4 servings per week

44) How many servings of butter or cream do you consume per week?
(A serving is 1 tablespoon butter or margarine, 2 tablespoons of cream, or half and half)

◯ >5 servings per week
◯ ≤ 5 servings per week

45) How many servings of beans do you consume per week?
(A serving is ¼ cup cooked beans, peas, chickpeas, or lentils, 2 ounces tofu)

◯ < 3 servings per week
◯ ≥ 3 servings per week

46) How many servings of whole grains do you consume per day?
(A serving is 1 slice bread, 1 small tortilla, ½ cup cooked grain like oats, kasha, bulgur, brown rice, pasta, 1 ounce dry pasta or rice, ½ cup shredded wheat, 1 cup ready to eat cereal flakes, 3 tablespoons wheat germ, 3 cups popped popcorn)

◯ < 3 servings per day
◯ ≥ 3 servings per day

47) How many servings of commercial sweets, candy bars, pastries, cookies, or cakes do you consume per week?
(A serving is 2 inch square brownie, 1 large or 3 small cookies, 1 energy or breakfast bar, 1 medium doughnut, 1 small cupcake, ½ cup ice cream, sherbet, or frozen yogurt, 1 small Danish)

◯ >4 servings per week
◯ ≤4 servings per week

48) How many servings of nuts do you consume per week?
(A serving is ¼ cup of nuts, 1 tablespoon of nut butter, 2 teaspoons of tahini or sesame paste, 1 tablespoon pumpkin or sunflower seeds)

◯ < 4 servings per week
◯ ≥ 4 servings per week

49) How many times per week do you consume meals from fast food restaurants?

◯ >1 serving per week
◯ ≤1 serving per week

50) How much alcohol do you drink per day?
(A serving is 5 ounces of wine, 12 ounces of beer, 1.5 ounces of spirits)

◯ >1 servings
◯ ≤ 1 serving

Pittsburgh Sleep Quality Index (PSQI)

Please complete the survey below to the best of your abilities.

Thank you!

INSTRUCTIONS:
The following questions relate to your usual sleep habits during the past month only. Your answers should indicate the most accurate reply for the majority of days and nights in the past month. Please answer all questions.

1. During the past month, what time have you usually gone to bed at night?	______________________ (BED TIME)
2. During the past month, how long (in minutes) has it usually taken you to fall asleep each night?	______________________ (NUMBER OF MINUTES)
3. During the past month, what time have you usually gotten up in the morning?	______________________ (GETTING UP TIME)
4. During the past month, how many hours of actual sleep did you get at night? (This may be different than the number of hours you spent in bed.)	______________________ (HOURS OF SLEEP PER NIGHT)

For each of the remaining questions, check the one best response. Please answer all questions.

5. During the past month, how often have you had trouble sleeping because you . . .

5a) Cannot get to sleep within 30 minutes	◯ Not during the past month<br>◯ Less than once a week<br>◯ Once or twice a week<br>◯ Three or more times a week
5b) Wake up in the middle of the night or early morning	◯ Not during the past month<br>◯ Less than once a week<br>◯ Once or twice a week<br>◯ Three or more times a week
5c) Have to get up to use the bathroom	◯ Not during the past month<br>◯ Less than once a week<br>◯ Once or twice a week<br>◯ Three or more times a week
5d) Cannot breathe comfortably	◯ Not during the past month<br>◯ Less than once a week<br>◯ Once or twice a week<br>◯ Three or more times a week
5e) Cough or snore loudly	◯ Not during the past month<br>◯ Less than once a week<br>◯ Once or twice a week<br>◯ Three or more times a week

5f) Feel too cold	◯ Not during the past month<br>◯ Less than once a week<br>◯ Once or twice a week<br>◯ Three or more times a week
5g) Feel too hot	◯ Not during the past month<br>◯ Less than once a week<br>◯ Once or twice a week<br>◯ Three or more times a week
5h) Had bad dreams	◯ Not during the past month<br>◯ Less than once a week<br>◯ Once or twice a week<br>◯ Three or more times a week
5i) Have pain	◯ Not during the past month<br>◯ Less than once a week<br>◯ Once or twice a week<br>◯ Three or more times a week
5j) Other reason(s), please describe	________________________________
How often during the past month have you had trouble sleeping because of this?	◯ Not during the past month<br>◯ Less than once a week<br>◯ Once or twice a week<br>◯ Three or more times a week
6. During the past month, how would you rate your sleep quality overall?	◯ Very good<br>◯ Fairly good<br>◯ Fairly bad<br>◯ Very bad
7. During the past month, how often have you taken medicine to help you sleep (prescribed or "over the counter")?	◯ Not during the past month<br>◯ Less than once a week<br>◯ Once or twice a week<br>◯ Three or more times a week
8. During the past month, how often have you had trouble staying awake while driving, eating meals, or engaging in social activity?	◯ Not during the past month<br>◯ Less than once a week<br>◯ Once or twice a week<br>◯ Three or more times a week
9. During the past month, how much of a problem has it been for you to keep up enough enthusiasm to get things done?	◯ No problem at all<br>◯ Only a very slight problem<br>◯ Somewhat of a problem<br>◯ A very big problem
10. Do you have a bed partner or room mate?	◯ No bed partner or room mate<br>◯ Partner/room mate in other room<br>◯ Partner in same room, but not same bed<br>◯ Partner in same bed

If you have a roommate or bed partner, ask him/her how often in the past month you have had...

10a) Loud snoring	◯ Not during the past month<br>◯ Less than once a week<br>◯ Once or twice a week<br>◯ Three or more times a week
10b) Long pauses between breaths while asleep	◯ Not during the past month<br>◯ Less than once a week<br>◯ Once or twice a week<br>◯ Three or more times a week
10c) Legs twitching or jerking while you sleep	◯ Not during the past month<br>◯ Less than once a week<br>◯ Once or twice a week<br>◯ Three or more times a week
10d) Episodes of disorientation or confusion during sleep	◯ Not during the past month<br>◯ Less than once a week<br>◯ Once or twice a week<br>◯ Three or more times a week
10e) Other restlessness while you sleep; please describe	______________________________
	◯ Not during the past month<br>◯ Less than once a week<br>◯ Once or twice a week<br>◯ Three or more times a week

Acceptability Questionnaire (AIM)

Please complete the final survey below.

Thank you!

---

77) The Oura ring meets my approval

- ◯ Completely disagree
- ◯ Disagree
- ◯ Neither agree nor disagree
- ◯ Agree
- ◯ Completely agree

---

78) The Oura ring is appealing to me

- ◯ Completely disagree
- ◯ Disagree
- ◯ Neither agree nor disagree
- ◯ Agree
- ◯ Completely agree

---

79) I like the Oura ring

- ◯ Completely disagree
- ◯ Disagree
- ◯ Neither agree nor disagree
- ◯ Agree
- ◯ Completely agree

---

80) I welcome the Oura ring

- ◯ Completely disagree
- ◯ Disagree
- ◯ Neither agree nor disagree
- ◯ Agree
- ◯ Completely agree

---

81) Additional comments related to use of the Oura ring

____

IPAQ Short

Please complete the survey below to the best of your abilities.

Thank you!

We are interested in finding out about the kinds of physical activities that people do as part of their everyday lives. The questions are about the time you spent being physically active in the last 7 days. Please answer each question even if you do not consider yourself to be an active person. In answering the following questions, vigorous physical activities refer to activities that take hard physical effort and make you breathe much harder than normal. Moderate activities refer to activities that take moderate physical effort and make you breathe somewhat harder than normal.

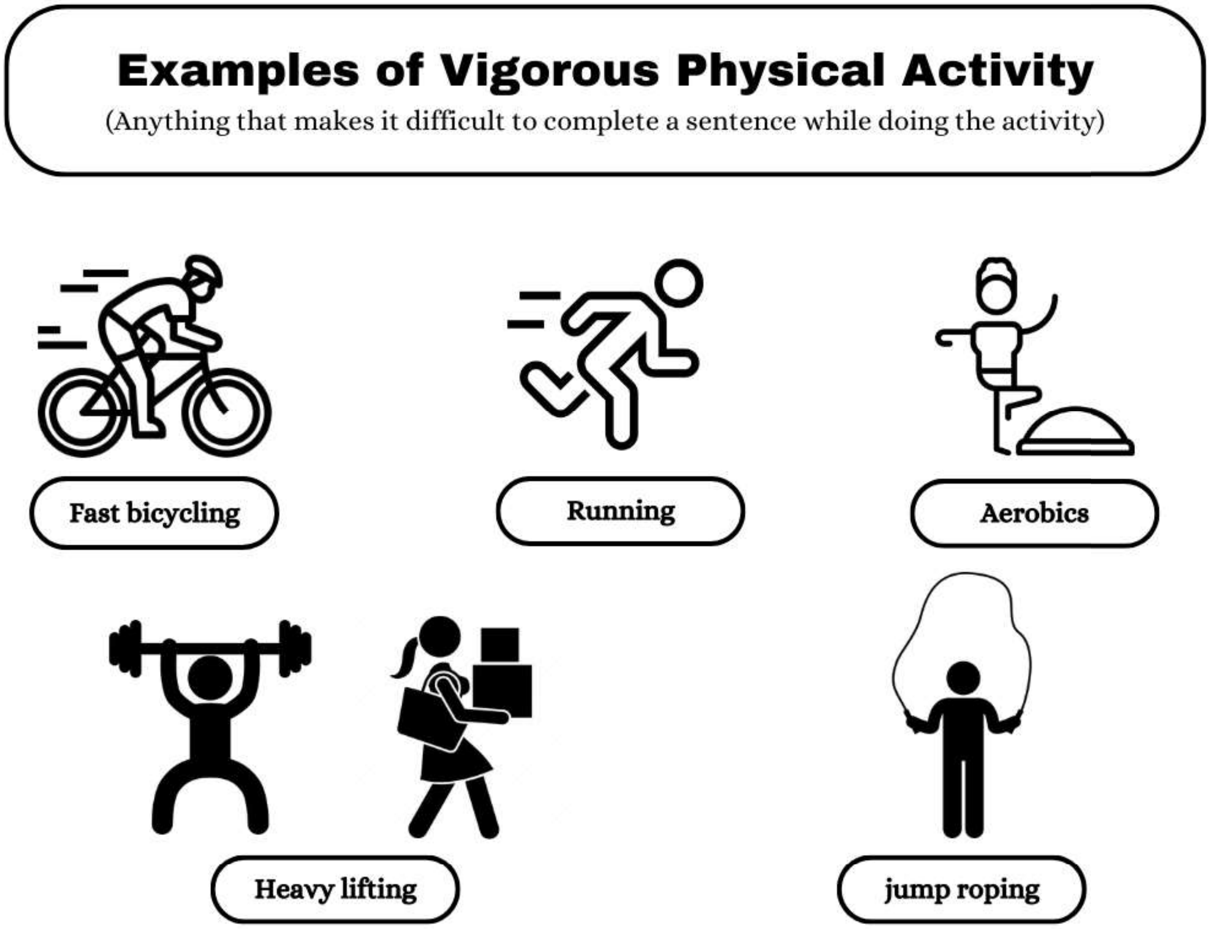

1a. During the last 7 days, on how many days did you do vigorous physical activities like heavy lifting, digging, aerobics, or fast bicycling?<br><br>Think about only those physical activities that you did for at least 10 minutes or more.	◯ 1 day per week<br>◯ 2 days per week<br>◯ 3 days per week<br>◯ 4 days per week<br>◯ 5 days per week<br>◯ 6 days per week<br>◯ 7 days per week<br>◯ none

1b. How much time in total did you usually spend on each of those days doing vigorous physical activities?

{ipaq_ss_vdhrs_v2} hours {ipaq_ss_vdmin_v2} minutes
On avergare PER day

(i.e. I ran for 15 minutes and lifted heavy weights for 20 minutes 3 days per week, so I would write down 0 hour and 35 minutes.)

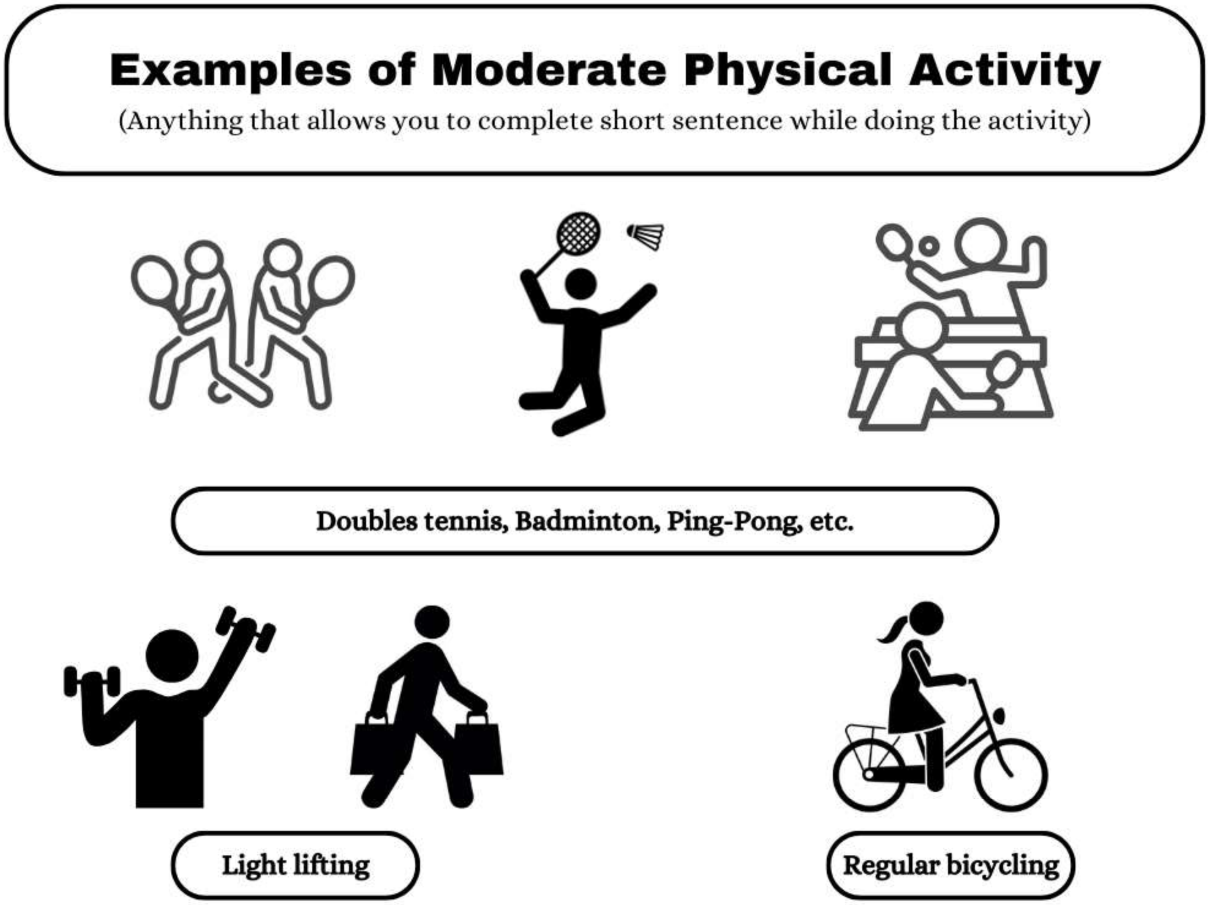

2a. Again, think only about those physical activities that you did for at least 10 minutes at a time. During the last 7 days, on how many days did you do moderate physical activities like carrying light loads, bicycling at a regular pace, or doubles tennis? Do not include walking.	◯ 1 day per week<br>◯ 2 days per week<br>◯ 3 days per week<br>◯ 4 days per week<br>◯ 5 days per week<br>◯ 6 days per week<br>◯ 7 days per week<br>◯ none

2b. How much time in total did you usually spend on each of those days doing moderate physical activities?

{ipaq_ss_mdhrs_v2} hours {ipaq_ss_mdmin_v2} minutes
On average PER day

(i.e. I carried groceries for 15 minutes 2 days per week, so I would write down 0 hour and 15 minutes.)

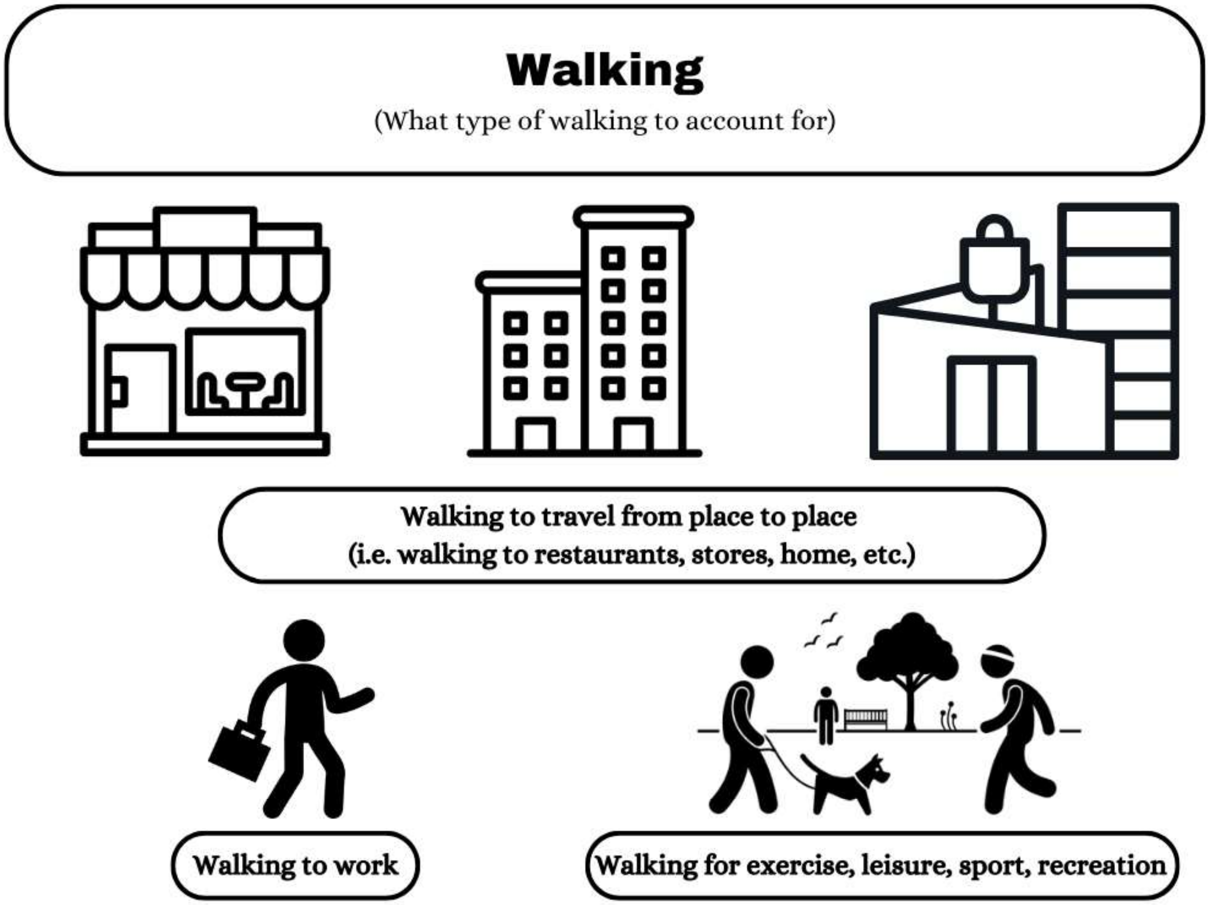

3a. During the last 7 days, on how many days did you walk for at least 10 minutes at a time? This includes walking at work and at home, walking to travel from place to place, and any other walking that you did solely for recreation, sport, exercise or leisure.

- ◯ 1 day per week
- ◯ 2 days per week
- ◯ 3 days per week
- ◯ 4 days per week
- ◯ 5 days per week
- ◯ 6 days per week
- ◯ 7 days per week
- ◯ none

3b. How much time in total did you usually spend walking on each of those days?

{ipaq_ss_wdhrs_v2} hours {ipaq_ss_wdmin_v2} minutes
On average PER day

(i.e. I walk 30 minutes every day to work, so in one day I walk a total of 1 hour for 5 days a week and stay home on the weekends. So, I would write down 1 hour and 0 minutes.)

The last question is about the time you spent sitting on weekdays while at work, at home, while doing course work and during leisure time. This includes time spent sitting at a desk, visiting friends, reading traveling on a bus or sitting or lying down to watch television.

4. During the last 7 days, how much time in total did you usually spend sitting on a week day?

{ipaq_ss_sdhrs_v2} hours {ipaq_ss_sdmin_v2} minutes
