# Supplementary material for: Sleep and Cardiovascular Health Among Women With a History of Hypertensive Disorders of Pregnancy: Pilot Observational Study
Source: JMIR Cardio. 2026 May 26;10:e81118. doi: 10.2196/81118 (PMC13211945; doi:10.2196/81118)
Supplement: Multimedia Appendix 3 [file cardio-v10-e81118-s003.docx]

**Supplemental Tables:**

**Table S1: Results of AIM questionnaire**

| **Questions** | **All (N=49)** |
| --- | --- |
| The Oura ring meets my approval, median (IQR) | 4 (4-5) |
| The Oura ring is appealing to me, median (IQR) | 4 (4-5) |
| I like the Oura ring, median (IQR) | 4 (4-5) |
| I welcome the Oura ring,  median (IQR) | 4 (4-5) |

**AIM scoring: completely disagree-1, disagree- 2, neither agree nor disagree- 3, agree-4, completely agree-5**

**Table S2: Cardiovascular Health (CVH) score and its components**

|  | **All (N=49)** | **HDP (N=28)** | **Normotensive (N= 21)** | **P-value** |
| --- | --- | --- | --- | --- |
| Overall CVH score | 70.7 ± 12.8 | 70.9 ± 11.4 | 70.3 ± 14.7 | .87 |
| Diet | 37.3 ± 25.6 | 41.1 ± 24.3 | 32.0 ± 26.9 | .16 |
| Physical activity | 76.1 ± 41.6 | 77.0 ± 42.1 | 74.7 ± 42.1 | .61 |
| Sleep | 72.6 ± 28.7 | 66.8 ± 30.7 | 76.2 ± 26.7 | .27 |
| Nicotine | 97.9 ± 7.0 | 97.3 ± 7.9 | 98.8 ± 5.6 | .52 |
| Body mass index (BMI) | 50.8 ± 35.4 | 54.1 ± 35.4 | 46.4 ± 35.9 | .46 |
| Lipids | 72.6 ± 28.6 | 77.0 ± 27.9 | 67.4 ± 29.2 | .83 |
| Glucose | 85.1 ± 22.7 | 88.7 ± 23.2 | 81.0 ± 22.0 | .66 |
| Blood pressure (BP) | 77.0 ± 30.1 | 70.4 ± 31.2 | 86.0 ± 26.8 | .07 |

**Table S3: Correlation between sleep health score and the other components of cardiovascular health**

| **Spearman’s Rank** |  |  |
| --- | --- | --- |
| **LE8 Component** | **Spearman’s ρ** | **p-value** |
| Physical Activity | −0.093 | .539 |
| Nicotine | 0.169 | .252 |
| BMI | 0.174 | .236 |
| Lipids | −0.136 | .397 |
| Blood Pressure | 0.046 | .756 |
| Blood Glucose | 0.124 | .434 |
| Diet | −0.002 | .989 |
